# Cortical volumetry and longitudinal cognitive changes in Parkinson’s disease: insights from the COPPADIS cohort

**DOI:** 10.1007/s11682-025-01031-8

**Published:** 2025-06-24

**Authors:** Tania Álvarez-Avellón, Carmen Solares, Juan Álvarez-Carriles, Pablo Franco-Rosado, Patricia Diaz-Galvan, Diego Santos-García, Pablo Mir, Manuel Menéndez-González

**Affiliations:** 1 Universidad de Oviedo, Oviedo, Spain; 2 Instituto de Investigación Sanitaria del Principado de Asturias, Oviedo, Spain; 3 Hospital Universitario Central de Asturias, Oviedo, Spain; 4 Unidad de Trastornos del Movimiento, Servicio de Neurología, Instituto de Biomedicina de Sevilla, Hospital Universitario Virgen del Rocío/CSIC/Universidad de Sevilla, Seville, Spain; 5 Complejo Hospitalario Universitario de A Coruña, A Coruña, Spain; 6 Grupo de Investigación en Enfermedad de Parkinson y Otros Trastornos del Movimiento, INIBIC (Instituto de Investigación Biomédica de A Coruña), A Coruña, Spain; 7 Hospital San Rafael, A Coruña, Spain; 8 Fundación Degen, A Coruña, Spain; 9 Centro de Investigación Biomédica en Red Sobre Enfermedades Neurodegenerativas (CIBERNED), Instituto de Salud Carlos III, Madrid, Spain; 10 Departamento de Psicología Experimental, Universidad de Sevilla, Seville, Spain; 11 Departamento de Medicina, Facultad de Medicina, Universidad de Sevilla, Seville, Spain

**Keywords:** Parkinson’s disease, Brain atrophy, Brain cortex, Cognitive performance, Cognitive impairment, Neuroanatomy

## Abstract

Cognitive decline is a major non-motor symptom in Parkinson’s disease (PD), often linked to brain atrophy. This study examines the relationship between cortical atrophy and age groups in predicting cognitive decline in PD over five years. 188 PD patients from the COPPADIS cohort were stratified by age: young (30–55 years, N = 47), middle-aged (56–65 years, N = 59), and older adults (66–75 years, N = 82). Baseline cortical volume was assessed using T1-weighted MRI, and cognitive decline was evaluated using the annual rate of change of the Parkinson’s Disease-Cognitive Rating Scale (PD-CRS). Parametric or non-parametric tests were applied to evaluate group differences. Main analyses consist of several multiple regression analyses to examine associations between baseline brain atrophy and cognitive decline by age group. Older adults exhibited significantly greater cognitive decline in comparison to the younger age groups in the three compose scores of the PDCRS -Fronto-subcortical (H(2) = 41.08, p < 0.001), Posterior Cortical (H(2) = 22.03, p < 0.001), and Total(H(2) = 41.13, p < 0.001). Higher educational level has a significant positive effect on older adults, specifically for working memory performance, delayed verbal memory and the fronto-subcortical composed score. Multiple regression models underscored the predictive value of the bilateral hippocampus, bilateral medial orbitofrontal cortex, right precuneus, and right isthmus cingulate gyrus, together with being an older adult or having a higher education. MRI measures, age, and education predict cognitive decline in PD. Longitudinal assessments are essential for refining atrophy-cognition correlations and optimizing patient stratification.

**Supplementary Information:**

The online version contains supplementary material available at 10.1007/s11682-025-01031-8.

## Introduction

Parkinson’s disease (PD) is a progressive neurodegenerative disorder marked by a constellation of motor and non-motor symptoms. While motor symptoms dominate the clinical picture, non-motor manifestations such as cognitive decline play a particularly significant role, affecting executive function, memory, attention, and visuospatial abilities. These deficits often coexist with mood disturbances, autonomic dysfunction, and sleep disorders, further complicating disease management and diminishing quality of life (Fang et al., 2020; Papagno & Trojano, 2018).

Cognitive decline in PD is highly heterogeneous, with trajectories influenced by genetic, environmental, and neuroanatomical factors (Bandres-Ciga et al., 2019; Carceles-Cordon et al., 2023). Longitudinal studies have characterized the prevalence and progression of dementia in PD, underscoring the variability in cognitive outcomes and the need for precise biomarkers to identify patients at higher risk (Aarsland et al., 2003; Puig-Davi et al., 2024). Mild cognitive impairment (MCI) in PD, an intermediate stage between normal cognition and dementia, has gained attention as a pivotal marker for early detection and intervention (Jellinger, 2024). However, despite its clinical relevance, the neuroanatomical underpinnings of MCI and its progression remain inadequately understood.

The cognitive deficits in PD are largely attributed to widespread cortical and subcortical changes, including atrophy and disrupted connectivity, which extend beyond dopaminergic pathways. Cortical regions such as the prefrontal cortex, hippocampus, and parietal areas are critically involved in these impairments, reflecting the disease’s multifaceted neuroanatomical impact (Braak et al., 2003; Brandão et al., 2024; Chaudhuri et al., 2005). Emerging research highlights the value of volumetric analyses in elucidating the relationship between structural changes and cognitive decline, yet much of this work has been cross-sectional. Several longitudinal MRI studies have previously explored the relationship between cortical atrophy and cognitive decline in Parkinson’s disease, highlighting regions such as the hippocampus, precuneus, orbitofrontal cortex, and basal forebrain as key contributors to cognitive trajectories (Jia et al., 2015; Mak et al., 2015; Pereira et al., 2020; Ray et al., 2018; Weintraub et al., 2012). These studies have provided valuable insights into brain-behavior relationships in PD.

However, most of them relied on general cognitive screening tools or focused on de novo patients with relatively short follow-up. In contrast, our study uses a disease-specific cognitive scale (PD-CRS), includes a large multicenter cohort followed for up to five years, and introduces a novel stratification by age and education level, helping to elucidate the moderating role of demographic factors in brain-cognition relationships.

Recent clustering approaches have further advanced the classification of PD subtypes, incorporating cortical atrophy and longitudinal cognitive data to delineate distinct trajectories (Iguanzo et al., 2024). Such insights underscore the potential of integrating neuroimaging and cognitive measures to refine patient stratification and optimize therapeutic strategies. Age plays a crucial role in cognitive decline among individuals with PD, influencing both the rate and severity of cognitive impairment. Studies have shown that patients with PD and cognitive impairment exhibit accelerated brain aging compared to those without cognitive impairment, with an increased predicted age difference of 7.08 years in gray matter and 8.82 years in white matter (Chen et al., 2024). These findings suggest that PD with cognitive impairment experience greater neurodegenerative changes, leading to faster declines in memory, executive function, and motor abilities. Similarly, Teipel et al. demonstrated that while idiopathic PD patients had only a 0.7-year brain-age gap compared to controls, their cognitive trajectories were more closely linked to Alzheimer’s disease-like rather than PD-specific atrophy patterns (Teipel et al., 2024). Taken together, these studies underscore the heterogeneous impact of aging on cognitive decline in PD.

The effect of education on brain atrophy remains unclear. On one hand, education has been proposed as a protective factor that enhances cognitive reserve, delaying cognitive decline in individuals with minimal neurodegeneration. Studies suggest that higher education is linked to slower cognitive decline in those with less brain atrophy, but this advantage is lost or even reversed as atrophy progresses, indicating a depletion of cognitive reserve (Mungas et al., 2018). On the other hand, large-scale longitudinal MRI studies have challenged this view, showing that while education is modestly associated with greater cortical volume cross-sectionally, it does not significantly influence the rate of brain atrophy over time (Nyberg et al., 2021). This discrepancy suggests that while education may buffer cognitive decline early in the disease process, its protective effect diminishes as neurodegeneration advances, highlighting the complex interaction between cognitive reserve and structural brain change.

In this context, the current study aims to investigate how baseline cortical volumetric measures are associated with and influence cognitive performance in PD patients over five years, using data from the COPPADIS cohort. By focusing on longitudinal changes in cognition, the study seeks to identify key cortical regions and demographic factors, such as age group and education, that predict cognitive trajectories. These findings are expected to enhance our understanding of the neuroanatomical correlates of cognitive decline in PD and inform personalized diagnostic and therapeutic approaches.

## Methods

### Participants

This study included 188 non-demented PD patients and 45 healthy controls from the COPPADIS-2015 (Cohort of Patients with Parkinson's Disease in Spain, 2015) cohort, a prospective, multicenter, non-interventional study aimed at investigating PD progression. Patients aged 30–75 years with idiopathic PD (UK Brain Bank criteria) and MMSE ≥ 26 were included. Exclusion criteria included inability to complete questionnaires, other disabling neurological or severe non-neurological diseases, chronic anemia/hyperuricemia, active treatment with continuous levodopa infusion, apomorphine, deep brain stimulation, participation in conflicting clinical trials, or inability to ensure long-term follow-up. The detailed description of the cohort has been previously reported (Santos-García et al., 2016). Participants for this analysis were a subset of the COPPADIS-2015 cohort from five centers who underwent baseline 3D T1-weighted structural MRI scans.

Patients with dementia, defined as a Mini-Mental State Examination (MMSE) score < 26 or the inability to perform basic activities of daily living, were excluded (Dubois et al., 2007). All participants provided written informed consent, and the study was approved by the local ethics committees of the participating centers.

To ensure a balanced distribution across the wide age range of participants (30–75 years), the sample was stratified into three distinct age groups: young adults (30–55 years), middle-aged adults (56–65 years), and older adults (66–75 years). This approach was motivated by two key considerations: first, the empirical distribution of our sample, which showed natural clustering around these age intervals; and second, this segmentation, aligned with previous studies (Blin et al., 2015; Gallagher et al., 2024; Quinn et al., 1987), facilitated a more even representation of the population within each group.

### Neuropsychological assessment

Cognitive performance was evaluated in the “on” dopaminergic medication state using the Parkinson’s Disease Cognitive Rating Scale (PD-CRS), a gold-standard cognitive screening tool designed specifically for patients with PD (Mungas et al., 2018; Teipel et al., 2024). The PD-CRS assesses multiple cognitive domains, including sustained attention, working memory, alternating and action verbal fluency, immediate and delayed free recall, naming, and visuospatial abilities such as drawing and copying a clock. In addition to individual item scores, the scale provides three composite scores: the fronto-subcortical score, which reflects executive functions and working memory; the posterior-cortical score, which measures visuospatial and memory abilities; and the total PD-CRS score, which offers a comprehensive overview of global cognitive functioning (Kulisevsky & Pagonabarraga, 2009; Pagonabarraga, et al., 2008).

PD-CRS scores of patients were measured at baseline (V0), at 48 months (V48M), and at 60 months (V60M). These assessments took place within a window of ± 3 months from the scheduled date relative to the inclusion date. Using these data, the rate of cognitive change in the patients was calculated for each of the PD-CRS measures. These rates of change represent the dependent variables or outcomes in our analyses *rates of cognitive change* were calculated as the difference between baseline and follow-up PD-CRS scores, divided by the interval in months.

$$Cognitive\; Change \;Rate=\frac{\left(Final \;PDCRS \;score -Baseline \;PDCRS \;score\right)}{\left(Baseline \;PDCRS \;score\;*\;Time \;(in \;months) \;between \;baseline \;and \;final \;scores\right)}$$

In the formula the final PDCRS scores correspond to the scores obtained in the last assessment which could have been conducted for each patient either at V48M or at V60M.

### Neuroimaging acquisition, processing, and analysis

#### MRI acquisition

Structural MRI data were acquired using 1.5 T or 3 T clinical scanners with high-resolution 3D T1-weighted sequences, achieving approximately 1 mm isotropic spatial resolution. Scanners and acquisition protocols varied across the five recruiting centers and were not harmonized. Each participant was scanned at their recruitment center. Although no scanner harmonization procedure (e.g., ComBat) was applied, all images were processed using the same volumetric pipeline to minimize segmentation variability. Center-specific MRI acquisition parameters are described in detail elsewhere (Grothe et al., 2021; Santos-García et al., 2023). The average time between MRI acquisition and cognitive assessment was 75 ± 67 days (IQR: 24–104 days).We implemented the processing pipeline for surface-based morphometry (SBM) within the Computational Anatomy Toolbox (CAT12, Jena University Hospital) running under the Statistical Parametric Mapping package (SPM12, Wellcome Trust Center for Neuroimaging) in Matlab R2018a (MathWorks, Natick, MA, USA). Scans were segmented into gray matter (GM), white matter, and cerebrospinal fluid.GM volumes for cortical regions of interest (ROIs) and the hippocampus were extracted, with total intracranial volume (TIV) calculated to account for individual differences in head size. To ensure consistency, volumes were normalized to TIV, providing reliable measures of cortical and hippocampal structures to explore cognitive trajectories in PD. Volumetric measures for cortical ROIs were based on the automated parcellation provided by the Desikan-Killiany atlas, as implemented in CAT12. Cortical regions of interest (ROIs) were selected according to previous data showing regional neuroanatomical correlates of cognitive performance in PD, particularly when assessed with the PD-CRS (Brandão et al., 2024). These regions included the rostral medial frontal cortex, caudal medial frontal cortex, superior temporal cortex, transverse temporal cortex, inferior parietal cortex, supramarginal gyrus, caudal anterior cingulate cortex, rostral anterior cingulate cortex, isthmus of the cingulate cortex, posterior cingulate cortex, insula, precuneus, fusiform cortex, medial orbitofrontal cortex, and paracentral cortex. Additionally, the hippocampus was included in the analyses due to its established role in cognitive performance (Panah et al., 2023; Sakamaki-Tsukita et al., 2024). Each of the 15 cortical ROIs was analyzed bilaterally, resulting in 30 cortical regions plus the bilateral hippocampus, for a total of 32 volumetric measures.

### Statistical analyses

The sample was divided into three age groups: young adults (30–55 years), middle-aged adults (56–65 years), and older adults (66–75 years). This categorization was based on both the observed distribution of participants in the COPPADIS cohort and prior literature suggesting age-related inflection points in neurocognitive aging (Blin et al., 2015). Stratification into discrete age groups allowed clinically meaningful comparisons across different adult life stages and improved interpretability of group differences in cognitive trajectories.

The distribution of all variables (dependent, independent, and covariates) was tested using the one-sample Kolmogorov–Smirnov test. Based on these results, parametric or non-parametric tests were applied to evaluate group differences. Cortical and hippocampal volumetric variables measured at baseline (V0) were used as independent variables in variance analyses and as predictors in the regression analyses. All regional volumes were normalized by dividing the individual's total intracranial volume (TIV) to account for head size variability. Several independent one-way Analysis of Variance (ANOVAs) were conducted to assess differences in volumetric measures and cognitive change rates across age groups.

To examine the joint effects of age and education on the rate of change on each PDRCRS score, we fitted a generalized linear model (GLM) including the main effects of age group and educational attainment, as well as their interaction. Age group was coded as a categorical variable with three levels (young adults, middle-aged adults, and older adults), using older adults (≥ 66 years) as the reference category. Educational attainment was coded similarly with three levels (primary education or less [reference], secondary education, and university education). The formula of the model was as follows: Yi = β0 + β1(Age group) + β2(Education) + β3(Age group × Education) + εi; where Yi represents the rate of change in PDRCRS scores for individual i, and ​εi is the error term. Both categorical predictors of age and educational attainment were dummy-coded, and maximum likelihood estimation with robust standard errors was used.

Bivariate correlation analyses were performed to explore associations between volumetric measures, cognitive change rates and covariables. Several multivariate regression analyses were conducted to identify longitudinal relationships between baseline volumetric measures and cognitive outcomes. For each rate of change in the PDCRS, two multiple regression analyses were conducted. The first regression included as predictors those cortical structures of interest that had significantly correlated with the rate of change in the PDCRS. The second multiple regression analysis was adjusted for the following covariates: gender, age group, educational level, and disease duration in months. Categorical covariates were dummy coded to be entered in the analysis. Atypical cases diagnosis was performed for each analysis (i.e., in case there were more than 1% of cases) to inspect whether potential outliers influence or modify significantly the results. In cases where the number of predictors and covariates in the model exceeded the sample size limitations, bidirectional elimination stepwise method was used to select variables for inclusion in the regression model. Regression models were conducted separately for cortical structures of the left and right hemispheres.

To control for the risk of Type I error due to multiple comparisons, appropriate p-value adjustments were applied depending on the statistical analysis. For all independent parametric and non-parametric ANOVAs used to compare clinical variables across age groups, Bonferroni correction was applied to account for the three pairwise comparisons conducted. For generalized linear models (GLMs) and multiple linear regression analyses, the Holm-Bonferroni method was used to adjust p-values. This method was selected because it is less conservative than the standard Bonferroni correction, thereby providing a balance between minimizing Type I error and reducing the likelihood of Type II error in models involving multiple predictors.

Data management was performed using RStudio (version 4.4.1.). Analyses were conducted using RStudio (version 4.4.1) and IBM SPSS Statistics (version 27). A p-value < 0.05 was considered statistically significant unless otherwise specified following correction for multiple comparisons.

## Results

### Demographic and clinical characteristics

Table 1 provides a detailed overview of the sample's characteristics, including differences in intracranial volumes, baseline and longitudinal total scores in the PD-CRS. Analyses on the level of education across groups revealed significant differences among the age groups: young adults had higher educational attainment compared to older adults (χ^2^(4, N = 188) = 26.645, p < 0.001), while no significant differences were observed in disease duration across the groups (F(2,185) = 1.439, p = 0.240), Non-Motor Symptoms (F(2,188) = 2,017, p = 0.365) or Sleep problems (F(2,188) = 2,092, p = 0.351) across the groups.

**Table 1:** Descriptive table of the patient sample

	PATIENTS			
Age group	Young adults (30–55 years old)	Middle-aged adults (56–65 years old)	Older adults (66–75 years old)	Total
N	47	59	82	188
Male	32	44	42	118
Female	15	15	40	70
Age (mean)	48.13 (5.7)	61.10 (3.06)	70.34 (2.45)	61.89 (9.637)
Male (mean age)	48.19 (4.9)	61.43 (3.015)	70.62 (2.35)	61.11 (9.47)
Female (mean age)	48 (7.53)	60.13 (3.09)	70.05 (2.54)	63.20 (9.663)
Primary studies	13	28	60	101
Secondary studies	17	16	13	46
University studies	17	15	9	41
Months of PD disease evolution (mean)	60.6 (45.07)	58.27 (48.63)	79.9 (66.16)	68.29 (56.87)
Total intracraneal volume (mean)	1395.57 (153.32)	1445.67 (171.47)	1410.21 (155.43)	1417.68 (160.50)
Non-Motor Symptoms-NMSS- (mean)	45.85 (39.08)	42.25 (38.52)	49.62 (38.52)	46.36 (38.04)
Parkinson’s Sleep Scale-PDSS- (mean)	116.20 (25.57)	121.44 (22.92)	119.89 (20.08)	119.45 (22.41)
PDCRS total score Basal visit (mean)	100.77 (13.83)	95.77 (11.80)	85.41 (12.81)	92.38 (14.28)
PDCRS total score 48 M visit (mean)	103.85 (13.16)	93.34 (14.39)	77.40 (23.01)	88.69 (21.37)
PDCRS total score 60 M visit (mean)	103 (10.97)	94.55 (14.85)	71.37 (27.98)	87.30 (24.56)

Note. Values in parentheses represent the standard deviations of the means. 48 M = 48 months visit; 60 M = 60 months visit. For PDCRS total score 48 M visit total N = 146 and for PDCRS total score 60 M visit total N = 133

#### Cortical volume and cognitive decline across age groups

Independent one-way ANOVAs were performed to evaluate differences in cortical and hippocampal volumetric measures across the three age groups. For cortical variables with non-normal distributions, the non-parametric Kruskal–Wallis test was applied. Significant differences were identified in most cortical structures, with the largest discrepancies observed between the youngest (30–55 years) and oldest (66–75 years) groups. However, four specific structures—the left caudal anterior cingulate cortex (F(2,185) = 2.776, p = 0.065), the left frontal pole (F(2,185) = 1.448, p = 0.238), the right frontal pole (H(2) = 4.405, p = 0.111), and the right medial orbitofrontal cortex (F(2,185) = 2.310, p = 0.102)—did not show significant differences between age groups.

To further explore these four volumetric variables and assess whether patients differed from age-matched controls, factorial ANOVAs were conducted for each structure. These analyses included two independent factors: group (control, patient) and age group, along with their interaction. Data on control participants (N = 45) were selected from the COPPADIS database study and age-matched with PD patients (see Table 4 in supplementary materials). While patients consistently exhibited lower cortical volumes compared to controls across all four structures, no statistically significant differences were detected at the group level (patients vs. controls), age group level, or in the interaction between these factors.

Age also had a significant impact on cognitive decline rates, with older adults experiencing more pronounced declines across PD-CRS domains (PD-CRS total score: H(2) = 41.13, p < 0.001), particularly in attention, memory, and visuospatial abilities (see Table 2 with the Kruskal–Wallis analysis results for the differences in PD-CRS domain scores across age groups). Kruskal–Wallis analyses confirmed significant differences across all PD-CRS domains between age groups, emphasizing the accelerated cognitive decline in older patients compared to younger and middle-aged groups. These findings highlight age-related heterogeneity in PD progression, especially in cognitive domains such as attention, working memory, and visuospatial tasks.

**Table 2:** Kruskal–Wallis Test Results for PD-CRS Change Rate Variables Across Age Groups

Cognitive Variable	Young adults (mean)	Middle-aged adults (mean)	Older adults (mean)	H(2)	p-value	Significant Group Differences	p-value comparisons
Immediate Verbal Memory	0.003472 (.0047)	0.000625 (.0055)	−0.002366 (.0063)	21.55	< 0.001	OA > YA	< 0.001
Confrontation Naming	0.000727 (.0033)	0.000321 (.0017)	−0.001155 (.0037)	20.66	< 0.001	OA > MA OA > YA	< 0.001 < 0.001
Sustained Attention	−0.000082 (.0021)	−0.001222 (.0031)	−0.005180 (.0072)	30.80	< 0.001	OA > MA OA > YA	< 0.001 < 0.001
Working Memory	−0.001361 (.0036)	−0.000401 (.0054)	−0.006732 (.0065)	32.37	< 0.001	OA > MA OA > YA	< 0.001 < 0.001
Spontaneous Clock Drawing	0.000213 (.0021)	−0.000064 (.0027)	−0.002950 (.0090)	12.84	0.002	OA > MA OA > YA	0.002 0.006
Clock Copy	0.000220 (.0015)	−0.000372 (.0018)	−0.003617 (.0076)	10.77	0.005	OA > YA	0.002
Delayed Verbal Memory	0.008080 (.0124)	0.003171 (.0164)	−0.003117 (.0119)	22.35	< 0.001	OA > YA MA > YA	< 0.001 0.009
Alternating Verbal Fluency	0.001294 (.0086)	0.000568 (.0079)	−0.003628 (.0061)	13.13	0.001	OA > MA OA > YA	0.006 0.001
Action Verbal Fluency	0.000016 (.00059)	0.000026 (.0045)	−0.003520 (.0070)	14.76	< 0.001	OA > MA OA > YA	0.001 0.002
Frontosubcortical	0.000543 (.0021)	−0.000273 (.0030)	−0.004279 (.0053)	41.08	< 0.001	OA > MA OA > YA	< 0.001 < 0.001
Posterior Cortical	0.000427 (.0016)	0.000053 (.0012)	−0.001991 (.0039)	22.03	< 0.001	OA > MA OA > YA	0.001 < 0.001
Total PD-CRS	0.000444 (.0015)	−0.000192 (.0021)	−0.003529 (.0045)	41.13	< 0.001	OA > MA OA > YA	< 0.001 < 0.001

**Note.** Means and Standard Deviation’s for PDCRS rate of change. *H represents the Kruskal–Wallis test statistic with 2 degrees of freedom. YA* = *Young adults; MA* = *Middle-aged adults; OA* = *Older adults. P-values for pairwise comparisons were adjusted using Bonferroni correction for 3 comparisons (adjusted significance threshold: p* < *0.0167).* In total, the number of patients for whom PDCRS rate of change could be calculated was 157 patients (Young adults N = 38; Middle-aged adults N = 50; Older adults N = 69)

**Figure 1:**
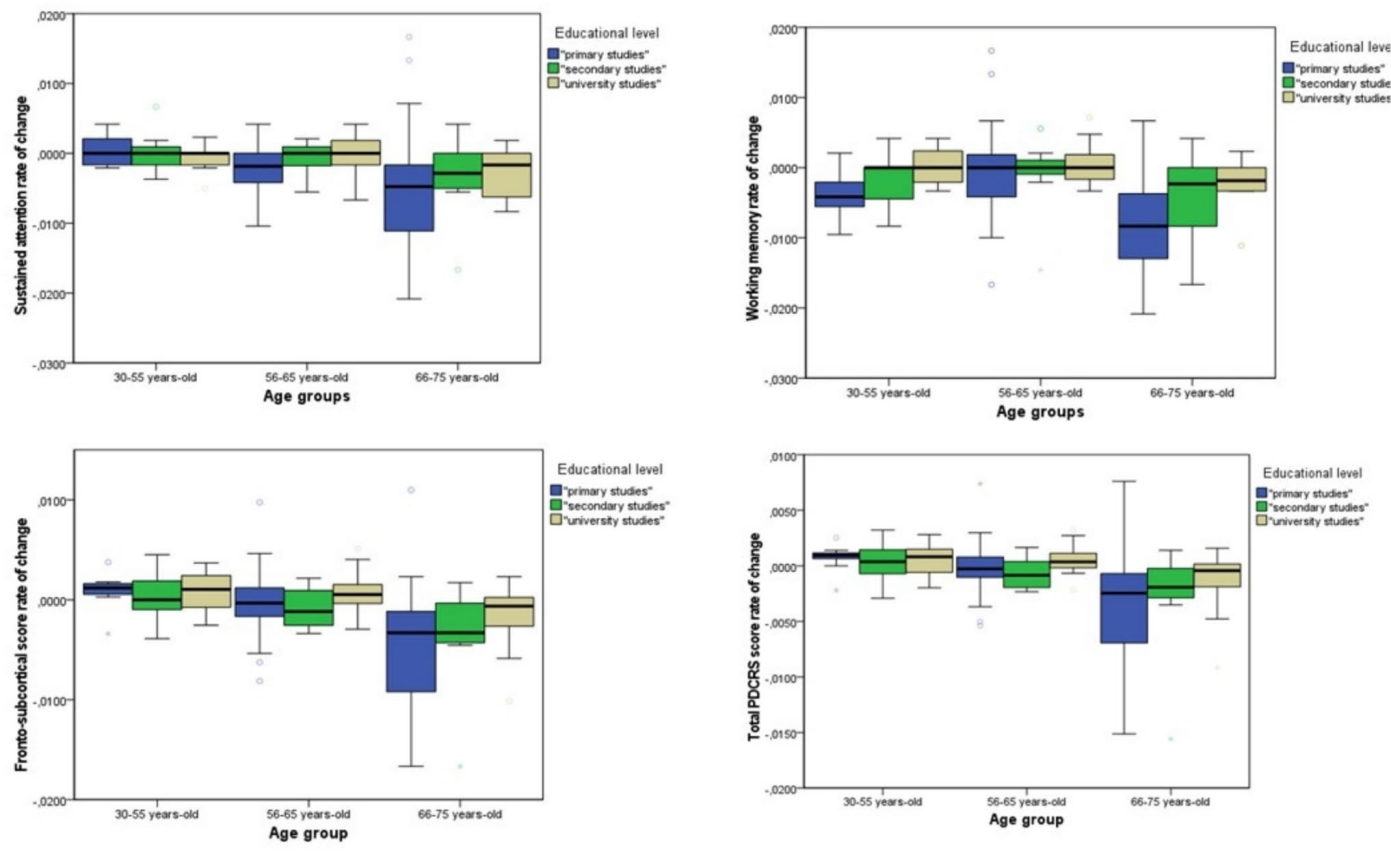
Visual representation of the association between the age group and the educational level for the rate of change of sustained attention, working memory, fronto-subcortical score and total PDCRS scores

Moreover, results from the GMLs showed a significant interaction between having a university education and being an older adult (66–75 years) on rates of change in sustained attention (χ2 = 6,096, df = 2, p = 0,047, β = 0.003, 95% CI [0.000, 0.006], p = 0.040). For working memory (χ2 = 2,553, df = 2, p > 0,05), delayed verbal memory (χ2 = 5,315, df = 2, p > 0,05), and the fronto-subcortical score (χ2 = 3,976, df = 2, p > 0,051) the interactions between age groups and educational levels did not reach statistical significance. However, the analysis of individual parameters revealed that having a university education has a significant positive effect on older adults, specifically for working memory performance (χ2 = 7,070, df = 1, β = 0.005, 95% CI [0.001, 0.008], p = 0.008),delayed verbal memory (χ2 = 5,589, df = 1, β = 0.013, 95% CI [0.002, 0.023], p = 0.018), and fronto-subcortical scores (χ2 = 4,166, df = 1, β = 0.003, 95% CI [0.000, 0.006], p = 0.041). These findings suggest that, within this group, a university education may enhance performance across these cognitive domains. Furthermore, having a university education had a significant overall effect on the rate of change in sustained attention (Wald χ^2^ = 4,859, df = 1, p < 0.05), working memory (Wald χ^2^ = 13.591, df = 1, p < 0.001), the fronto-subcortical score (χ2 = 4,986, df = 1, p < 0,05) and the total PD-CRS score (Wald χ^2^ = 4.305, df = 1, p < 0.05) (Fig. 1).

## Volumetric correlations with cognitive change and models for cognitive change

### Bivariate correlations

Significant correlations were observed between specific cortical regions and the total PD-CRS scores, as well as other domain-specific scores. These correlations varied across hemispheres, highlighting the importance of laterality in predicting cognitive trajectories (see supplementary materials, Table 1). In the right hemisphere, the strongest correlations (above r = 0.300) with the total PD-CRS score were observed for the hippocampus (r = 0.497, p < 0,001), precuneus (r = 0.398, p < 0,001), and isthmus cingulate gyrus (r = 0.407, p < 0,001). These regions also exhibited robust associations with subdomains such as attention (hippocampus: r = 0.418, p < 0,001; precuneus: r = 0.313, p < 0,001) and visuospatial abilities (isthmus cingulate: r = 0.327, p < 0,001). In the left hemisphere, the hippocampus (r = 0.427, p < 0,001), fusiform cortex (r = 0.395, p < 0,001), and precuneus (r = 0.332, p < 0,001) demonstrated strong correlations with the total PD-CRS scores.

### Multiple regression models

Multiple regression models were employed to determine significant longitudinal association between baseline volumetric measures and cognitive outcomes. Table 3 shows the main results for the three composed scores of the PD-CRS (i.e., frontal subcortical score, posterior cortical score, total PD-CRS score). A more detailed summary of the results for all the cognitive measures are reported in the supplemental materials (Tables 2 & 3).

**Table 3:** Main results of the multiple linear regression models to study the influence of left and right cortical volumes on the different composed measures of the PDCRs

		Model 1			Model 2		
		B	95% CI	β	B	95% CI	Β
	Left Hemisphere						
Fronto-subcortical score ^&^	Hippocampus	0.022	0.014, 0.030	0.276**	0.015	0.007, 0.022	0.271**
	Medial Orbitofrontal	0.015	0.004, 0.027	0.198*	0.013	0.003, 0.024	0.169*
	Older Adults				−0.003	−0.005, −0.002	−0.347**
	R-squared		0.217***			0.316***	
	Adjusted R-squared		0.207			0.302	
Posterior-cortical score ^&^	Hippocampus				0.007	0.002, 0.013	0.204**
	Older Adults				−0.002	−0.003, −0.001	−0.295**
	R-squared		0.177*			0.180***	
	Adjusted R-squared		0.126			0.169	
Total PD-CRS score^&^	Hippocampus	0.017	0.010, 0,023	0.376**	0.012	0.005, 0.018	0.270**
	Medial Orbitofrontal	0.012	0.002, 0,021	0.175*	0.011	0.002, 0.020	0.165*
	Transversetemporal	0.024	0.000, 0,047	0.146*			
	Older Adults				−0.003	−0.004, −0.001	−0.342**
	R-squared		0.237***			0.313**	
	Adjusted R-squared		0.222			0.299	
	Right Hemisphere						
Fronto-subcortical score ^&^	Hippocampus	0.018	0.010, 0.026	0.370**	0.013	0.005, 0.021	0.262**
	Isthmuscingulate	0.030	0.010, 0.051	0.236**	0.025	0.006, 0.045	0.196*
	Older Adults				−0.002	−0.004, −0.001	−0.263**
	University Education				0.002	0.000, 0.003	0.155*
	R-squared		0.279***			0.372***	
	Adjusted R-squared		0.270			0.355	
Posterior-cortical score ^&^	Hippocampus	0.011	0.005, 0.018	0.359*	0.010	0.004, 0.015	0.298**
	Older Adults				−0.001	−0.002, 0.000	−0.240*
	R-squared		0.207**			0.214***	
	Adjusted R-squared		0.141			0.204	
Total PD-CRS score^&^	Hippocampus	0.016	0.009, 0,022	0.389**	0.011	0.005, 0.018	0.280**
	Isthmuscingulate	0.024	0.006, 0,041	0.220*	0.020	0.003, 0.036	0.184*
	Older Adults				−0.002	−0.003, −0.001	−0.257**
	University Education				0.001	0.000, 0,003	0.142*
	R-squared		0.285***			0.369***	
	Adjusted R-squared		0.275			0.352	

Note: The table only presents the coefficients and values for the independent variables and covariates that showed a significant effect in the model. If no independent variable contributed significantly to the model, but the overall model was significant, or if the model was not significant, only the R^2^ and adjusted R^2^ values are reported; Model 1: Multiple linear regression model including only the cortical volumes relevant to each analysis; Model 2: Model adjusted for the covariates: age group, educational level, and disease duration; & = The independent variables and covariates were entered into the model using the Stepwise method. Stepwise method was used in the following analyses of the left hemisphere structures: Model 1 and 2 for the frontosubcortical score; Model 2 for the posterior cortical score; Models 1 and 2 for the total PDCRS score. Stepwise method was used for the following analyses of the right hemisphere structures: Model 2 for the posterior cortical score; Models 1 and 2 for the frontosubcortical score; Models 1 and 2 for the total PDCRS score. P-values were adjusted for multiple comparisons using the Holm-Bonferroni correction. * p < 0.05, ** p < 0.01, *** p < 0.001

Regression models further underscored the predictive value of the hippocampus across both hemispheres. For the right hemisphere, the full adjusted model explained around 35% of the variance in the total PD-CRS score, F (4, 152) = 22,133, p < 0,001, where the hippocampus was the most significant predictor (β = 0.280, t = 3,531, p < 0.01), together with the isthmus cingulate (β = 0.184, t = 2,440, p < 0.05), being an older adult (β = −0.257, t = 3,439, p < 0,01) and having a university education (β = 0.142, t = 2.110, p < 0,05). This pattern of results was consistent with other domains such as attention, R^2^ = 0,251, F (3, 151) = 16,882, p < 0,001 or the fronto subcortical score, R^2^ = 0,369, F (4, 152) = 22,216, p < 0,001, where the hippocampus also emerged as the most significant predictor (for attention: β = 0.315, t = 3,952, p < 0.01; for fronto-subcortical score: β = 0.262, t = 3,224, p < 0.01). The isthmus cingulate, having a high education level or being an older adult, also showed significant contributions as predictors (see Table 3 and supplemental Table 3).

In the left hemisphere, the full adjusted model explained around 31% of the variance in the total PD-CRS score, F (3, 153) = 23,228, p < 0,001, where the hippocampus remained a robust predictor for total PD-CRS (β = 0.270, t = 3,651, p < 0.01), together with the medial orbitofrontal cortex (β = 0.165, t = 2,454, p < 0.05) and being an older adult (β = −0,342, t = −4,619, p < 0.01). The full adjusted models performed for outcomes such as delay verbal memory (R^2^ = 0,150, F (3, 148) = 8,687, p < 0,001) and the fronto-subcortical score (R^2^ = 0,316, F (3, 147) = 22.681, p < 0,001) also showed that the hippocampus, the medial orbitofrontal cortex, being an older adult or having a high educational level contributed as significant predictors for these cognitive outcomes (Also see Table 3 and supplemental Table 2).

Few atypical cases were identified for some of the analysis (less than 3% of the cases in all the performed analysis), therefore we run sensitivity analysis where atypical cases were excluded from the regression models to explore their influence in the regression models. Sensitivity analysis revealed that running the regression analysis without these values did not change significantly the main results which confirmed that these atypical cases were not influencing significantly the regression models (see tables 5 & 6 in the supplemental material).

To ensure our findings are held under different conditions, we conducted sensitivity analyses in which age was entered as a continuous variable in all adjusted regression models. The results of these analyses showed that our findings remain consistent across different modeling strategies, thereby enhancing the robustness of our results (see tables 7 & 8 in the supplemental material).

## Discussion

This study investigated the relationship between cortical and hippocampal volumetric measures and cognitive performance in PD patients over five years, highlighting the role of structural changes in predicting long-term cognitive trajectories. The absolute magnitude of the rate of decline, although statistically significant, may be considered modest in clinical terms. Regarding structural changes, key regions —including bilateral hippocampus, bilateral medial orbitofrontal cortex, right precuneus, and right isthmus cingulate gyrus— emerged as significant predictors of cognitive decline. Our findings emphasize the importance of hemispheric laterality in assessing the impact of cortical atrophy on cognitive function. Specifically, left hemisphere structures, such as the hippocampus and fusiform cortex, were strongly associated with memory and overall cognitive performance, while right hemisphere regions, including the precuneus and isthmus cingulate gyrus, were more closely linked to visuospatial and executive functions.

Age emerged as a significant determinant of cognitive decline, with older adults exhibiting greater deficits in attention, working memory, and visuospatial abilities, as assessed by the PD-CRS. These findings align with previous studies demonstrating that neurodegenerative processes in PD accelerate brain aging and alter cognitive trajectories (Blin et al., 2015; Chen et al., 2024; Gallagher et al., 2024; Quinn et al., 1987; Teipel et al., 2024). For instance, Gallagher et al. found that by age at disease diagnosis, 40% among those who were younger than 56 years were diagnosed with dementia, while this percentage increases to 44.6% among those aged 56–70 years, and 61% among those older than 70 years (Gallagher et al., 2024). While younger PD patients displayed relative cognitive stability, older adults exhibited patterns of decline consistent with prior research highlighting working memory and motor sequence learning deficits as key contributors to age-related cognitive impairment (Santos-García et al., 2023). Our results also support the use of brain-age gap as a structural marker of PD progression. Previous studies have shown that PD patients with cognitive impairment (PD-CI) exhibit significantly larger brain-age gaps (7.08 years in gray matter, 8.82 years in white matter) compared to those without cognitive impairment (Mungas et al., 2018). Similarly, Teipel et al. found that while idiopathic PD patients had a modest 0.7-year brain-age gap compared to controls, their cognitive decline followed Alzheimer’s disease-like patterns rather than those typical of PD (Teipel et al., 2024). Stratifying patients by age revealed distinct cognitive trajectories. Younger patients exhibited slower cognitive decline compared to older patients, with consistently higher PD-CRS total and subdomain scores across all visits. No significant differences were observed between younger PD patients and age-matched controls, which is consistent with prior findings from the COPPADIS cohort demonstrating that mild cognitive impairment (MCI) and dementia are more prevalent in late-onset PD (LOPD) compared to young-onset PD (YOPD) (Santos-García et al., 2016).

Educational attainment significantly influenced cognitive trajectories, with higher education levels associated with better outcomes in working memory and fronto-subcortical tasks, reinforcing the protective role of cognitive reserve. These results align with prior studies demonstrating that greater educational attainment correlates with better cognitive function and a reduced risk of dementia, even in the presence of neurodegenerative disease (Cohen et al., 2007; Kotagal et al., 2015). Cohen et al. found that PD patients with higher education levels performed better on neuropsychological tests, particularly those assessing frontal lobe function, and exhibited a lower risk of hallucinations and neuropsychiatric symptoms (Cohen et al., 2007). Similarly, Kotagal et al. reported that higher education was associated with lower motor impairment severity and reduced white matter hyperintensities, suggesting a broader neuroprotective role extending beyond cognition (Kotagal et al., 2015).

Our study further highlights the importance of hemispheric laterality in cognitive decline, demonstrating that left hemisphere structures—particularly the hippocampus and medial orbitofrontal cortex—were strongly associated with memory and attention, while right hemisphere structures, including the isthmus cingulate gyrus and precuneus, were more predictive of visuospatial and fluency-related tasks. These findings extend prior research by emphasizing the differential impact of cortical atrophy across cognitive domains, suggesting that volumetric changes in specific brain regions contribute to distinct cognitive trajectories in PD. In this regard, previous studies have identified the basal forebrain, including the cholinergic nucleus basalis of Meynert, as strongly associated with cognitive decline in PD, potentially preceding neocortical involvement in some trajectories (Iguanzo et al., 2024; Jia et al., 2015; Mak et al., 2015; Pereira et al., 2020; Ray et al., 2018; Sakamaki-Tsukita et al., 2024; Weintraub et al., 2012). However, our MRI processing pipeline was optimized for cortical structures and did not include a specific parcellation of the basal forebrain. To ensure internal consistency across all analyzed regions, we opted not to derive composite or indirect measures for this area. Nevertheless, the inclusion of basal forebrain volumetry represents an important avenue for future research, especially given its key role in cholinergic signaling and early neurodegenerative changes in PD.

Bivariate correlations revealed that the hippocampus, precuneus, and medial orbitofrontal cortex were strongly associated with cognitive performance, particularly in older adults. Regression analyses confirmed these regions as robust predictors of cognitive decline, independent of disease duration, age, gender, or education. The hippocampus was a key predictor of attention, working memory, and delayed verbal memory, while the medial orbitofrontal cortex was closely tied to visuospatial abilities. These findings align with the dual-syndrome hypothesis, which attributes cognitive impairments in PD to frontostriatal dopaminergic dysregulation and cortical degeneration, each contributing to distinct cognitive deficits (Sakamaki-Tsukita et al., 2024). Specifically, Yoshimura et al. identified cortical thinning in frontotemporal and occipitoparietal regions as a hallmark of cognitive impairment in PD, supporting our findings that precuneus and posterior cortical atrophy predict early cognitive decline (Yoshimura et al., 2025). Similarly, frontotemporal thinning observed in PD-MCI aligns with our findings on the medial orbitofrontal cortex's role in cognitive decline. These results suggest that cortical changes in specific regions could serve as biomarkers for early cognitive impairment, reinforcing the need for volumetric measures in diagnostic and monitoring protocols.

Our findings emphasize the importance of integrating volumetric and demographic factors into patient assessments, allowing for more tailored strategies to mitigate cognitive decline in PD. Educational attainment emerged as a critical modulator, potentially enhancing neural plasticity and compensatory mechanisms. These findings support the implementation of cognitive training and lifelong learning programs to strengthen cognitive reserves. Additionally, age-specific strategies should be considered: younger patients may benefit from interventions that enhance compensatory mechanisms, while older patients require approaches targeting accelerated atrophy and cognitive decline.

This study has several limitations. First, cognitive assessment was limited to the PD-CRS, which provides item-level and partial score analyses but lacks the granularity of comprehensive neuropsychological batteries. Second, while cognitive performance was assessed longitudinally, we did not obtain repeated MRI scans, which precludes direct tracking of cortical volume changes over time. Third, although our regression models were robust, some variables (e.g., confrontation naming, clock drawing, clock copying, and the posterior cortical score) exhibited non-normally distributed residuals. Nevertheless, prior research suggests that linear regression remains reliable in large samples like ours (Schmidt & Finan, 2018). Fourth, different MRI scanners were used across recruitment centers. Although all images were processed using a standardized pipeline and regional volumes were normalized to intracranial volume, there was no harmonization of acquisition parameters between sites. Scanner allocation was determined by recruitment site and was not related to clinical variables such as age or disease severity. However, we acknowledge that the lack of harmonization may introduce site-related variance. Future studies may benefit from applying harmonization methods such as *ComBat* to further reduce inter-scanner variability and improve robustness of volumetric comparisons. Finally, a relevant consideration in the context of cognitive decline in PD is the potential contribution of amyloid and tau co-pathology. These biomarkers were not assessed in the COPPADIS cohort, and we were therefore unable to account for their influence on cognitive trajectories. However, emerging evidence indicates that co-pathologies may accelerate cognitive decline in PD and contribute to phenotypic heterogeneity (Irwin et al., 2017). Future research combining volumetric MRI with CSF or PET-based biomarkers of amyloid and tau would offer a more comprehensive understanding of disease mechanisms and progression.

Future studies should include larger and more age-representative control groups to enable robust comparisons between PD patients and healthy individuals. Further exploration of the interplay between cholinergic system integrity, hippocampal pathology, and cortical atrophy will be crucial for refining patient stratification and developing personalized therapeutic approaches.

## Conclusions

Cortical volumetry identifies key regions—hippocampus, medial orbitofrontal cortex, precuneus, and isthmus cingulate gyrus—as critical for understanding cognitive decline in PD. The hippocampus consistently emerged as the strongest predictor of cognitive decline across multiple domains, while the medial orbitofrontal cortex contributed specifically to working memory and visuospatial tasks. Notably, the precuneus and both hippocampi showed a robust predictive role in attention and memory deterioration, reinforcing their central role in cognitive aging in PD. Laterality plays a significant role, with left hemisphere structures predominantly associated with memory and attention, while right hemisphere regions are more closely linked to visuospatial and executive functions.

These findings underscore the necessity of considering age, longitudinal trajectories, and anatomical laterality when assessing cognitive decline in PD. While atrophy patterns provide valuable predictive insights, comprehensive longitudinal cognitive assessments remain essential for establishing stronger correlations between structural degeneration and cognitive deterioration. Future research integrating multimodal neuroimaging and longitudinal cognitive profiling will be critical for enhancing early detection, refining patient stratification, and advancing personalized therapeutic interventions in PD.

## Supplementary Information

Below is the link to the electronic supplementary material.

Supplementary file1 (DOCX 115 KB)

## Data Availability

Data supporting the findings of this study originates from the COPPADIS study and are available from the study Principal Investigator (DSG) upon reasonable request.
