# Supplementary material for: Cortical volumetry and longitudinal cognitive changes in Parkinson’s disease: insights from the COPPADIS cohort
Source: Brain Imaging Behav. 2025 Jun 24;19(5):1048–60. doi: 10.1007/s11682-025-01031-8 (PMC12518433; doi:10.1007/s11682-025-01031-8)
Supplement: Supplementary file 1 — Supplementary file1 (DOCX 115 KB) [file 11682_2025_1031_MOESM1_ESM.docx]

Supplementary table 1. Bivariate correlations between change rates in PDCRS variables, all anatomical cortical regions on the right and left hemisphere, and covariates.

|  | Immediate_verbal_memory | Confrontation_naming | Sustained_attention | Working_memory | Spontaneous_clock_drawing | Clock_copy | Delayed_verbal_memory | Alternating_verbal_fluency | Action_verbal_fluency | Frontosubcortical_score | Posterior_cortical_score | Total_pd_crs_score |
| --- | --- | --- | --- | --- | --- | --- | --- | --- | --- | --- | --- | --- |
| Time of disease evolution | -0,119 | -,188^*^ | -0,057 | -0,083 | 0,032 | -0,148 | -0,113 | -,157^*^ | -0,062 | -0,133 | -,205^**^ | -0,153 |
| MAA | 0,069 | 0,117 | ,175^*^ | ,327^**^ | 0,128 | ,161^*^ | 0,071 | 0,149 | ,171^*^ | ,234^**^ | ,182^*^ | ,236^**^ |
| OA | -,340^**^ | -,256^**^ | -,391^**^ | -,466^**^ | -,232^**^ | -,319^**^ | -,292^**^ | -,292^**^ | -,282^**^ | -,473^**^ | -,360^**^ | -,472^**^ |
| Sec. Edu. | 0,026 | 0,077 | 0,155 | 0,118 | 0,054 | 0,141 | 0,002 | 0,044 | -0,056 | 0,066 | 0,125 | 0,076 |
| Univ. Edu. | 0,134 | 0,046 | ,172^*^ | ,252^**^ | 0,105 | 0,127 | ,234^**^ | 0,126 | ,209^**^ | ,234^**^ | 0,119 | ,220^**^ |
| Gender | -,196* | -0,083 | -,241** | -0,135 | -0,105 | -0,083 | -,196* | -0,037 | -0,127 | -,172* | -0,101 | -,165* |
| Right Hemisfere Areas |  |  |  |  |  |  |  |  |  |  |  |  |
| Hippocampus | ,339^**^ | ,207^**^ | ,418^**^ | ,335^**^ | ,297^**^ | ,449^**^ | ,291^**^ | ,241^**^ | ,293^**^ | ,484^**^ | ,415^**^ | ,497^**^ |
| Caudalanteriorcingulate | ,260^**^ | 0,090 | ,178^*^ | ,191^*^ | ,194^*^ | ,241^**^ | ,170^*^ | ,204^*^ | ,193^*^ | ,285^**^ | ,208^**^ | ,288^**^ |
| Caudalmiddlefrontal | 0,078 | -0,017 | 0,151 | 0,106 | 0,077 | 0,142 | 0,113 | ,244^**^ | 0,153 | ,205^**^ | 0,094 | ,194^*^ |
| Cuneus | 0,089 | 0,074 | 0,035 | 0,031 | 0,038 | 0,118 | 0,105 | 0,103 | -0,085 | 0,058 | 0,123 | 0,077 |
| Entorhinal | ,227^**^ | ,220^**^ | ,271^**^ | ,284^**^ | ,232^**^ | ,336^**^ | ,262^**^ | ,186^*^ | ,214^**^ | ,344^**^ | ,347^**^ | ,363^**^ |
| Fusiform | 0,155 | ,176^*^ | ,283^**^ | ,302^**^ | ,183^*^ | ,274^**^ | ,233^**^ | ,276^**^ | ,182^*^ | ,339^**^ | ,286^**^ | ,346^**^ |
| Inferiorparietal | 0,146 | 0,052 | ,260^**^ | ,162^*^ | ,206^**^ | ,244^**^ | ,242^**^ | ,268^**^ | ,222^**^ | ,330^**^ | ,200^*^ | ,321^**^ |
| Inferiortemporal | 0,083 | 0,066 | ,193^*^ | 0,157 | 0,132 | ,196^*^ | 0,153 | ,223^**^ | ,192^*^ | ,263^**^ | ,178^*^ | ,265^**^ |
| Isthmuscingulate | ,344^**^ | 0,080 | ,337^**^ | ,207^**^ | ,237^**^ | ,327^**^ | ,312^**^ | ,206^**^ | ,302^**^ | ,417^**^ | ,259^**^ | ,407^**^ |
| Lateraloccipital | 0,072 | 0,009 | 0,142 | 0,143 | 0,002 | 0,076 | 0,155 | ,250^**^ | ,228^**^ | ,229^**^ | 0,057 | ,204^*^ |
| Lateralorbitofrontal | 0,111 | 0,042 | 0,154 | ,288^**^ | 0,127 | ,164^*^ | 0,130 | ,168^*^ | ,186^*^ | ,250^**^ | ,158^*^ | ,250^**^ |
| Lingual | ,159^*^ | 0,040 | 0,114 | 0,153 | 0,098 | 0,145 | 0,136 | ,184^*^ | 0,067 | ,202^*^ | 0,127 | ,199^*^ |
| Medialorbitofrontal | 0,052 | 0,073 | 0,111 | ,227^**^ | 0,133 | 0,103 | 0,041 | ,185^*^ | ,168^*^ | ,212^**^ | 0,127 | ,208^**^ |
| Middletemporal | 0,128 | 0,109 | ,194^*^ | ,232^**^ | 0,128 | ,259^**^ | ,228^**^ | ,260^**^ | ,193^*^ | ,302^**^ | ,235^**^ | ,311^**^ |
| Parahippocampal | ,267^**^ | 0,112 | ,264^**^ | 0,134 | 0,088 | ,304^**^ | ,282^**^ | 0,153 | 0,036 | ,257^**^ | ,256^**^ | ,277^**^ |
| Paracentral | 0,090 | 0,098 | 0,039 | 0,129 | -0,046 | 0,046 | 0,013 | ,220^**^ | 0,058 | 0,131 | 0,094 | 0,128 |
| Parsopercularis | ,251^**^ | 0,048 | ,176^*^ | ,273^**^ | 0,110 | ,193^*^ | 0,125 | ,272^**^ | ,174^*^ | ,311^**^ | ,164^*^ | ,299^**^ |
| Parsorbitalis | -0,044 | 0,007 | 0,110 | 0,144 | 0,037 | 0,142 | 0,106 | 0,133 | 0,144 | 0,133 | 0,095 | 0,132 |
| Parstriangularis | 0,035 | 0,023 | -0,002 | 0,156 | -0,015 | 0,122 | 0,138 | ,157^*^ | 0,056 | 0,118 | 0,105 | 0,124 |
| Pericalcarine | 0,127 | 0,035 | 0,017 | 0,129 | 0,079 | 0,128 | 0,078 | 0,119 | 0,066 | 0,136 | 0,111 | 0,144 |
| Postcentral | 0,043 | -0,019 | ,167^*^ | 0,142 | 0,098 | ,223^**^ | 0,137 | 0,145 | 0,114 | ,190^*^ | 0,139 | ,193^*^ |
| Posteriorcingulate | ,276^**^ | 0,043 | ,311^**^ | ,190^*^ | ,201^*^ | ,291^**^ | ,238^**^ | ,289^**^ | ,197^*^ | ,356^**^ | ,208^**^ | ,346^**^ |
| Precentral | 0,096 | 0,061 | 0,146 | ,168^*^ | 0,013 | ,163^*^ | ,175^*^ | ,303^**^ | ,177^*^ | ,239^**^ | 0,143 | ,227^**^ |
| Precuneus | ,287^**^ | 0,126 | ,313^**^ | ,238^**^ | ,222^**^ | ,317^**^ | ,295^**^ | ,302^**^ | ,224^**^ | ,396^**^ | ,288^**^ | ,398^**^ |
| Rostralanteriorcingulate | ,178^*^ | 0,121 | ,220^**^ | ,162^*^ | ,160^*^ | ,221^**^ | 0,075 | ,166^*^ | ,221^**^ | ,259^**^ | ,214^**^ | ,267^**^ |
| Rostralmiddlefrontal | -0,054 | -0,013 | 0,105 | 0,054 | -0,013 | 0,139 | 0,085 | 0,104 | 0,073 | 0,086 | 0,078 | 0,092 |
| Superiorfrontal | 0,109 | 0,150 | 0,108 | 0,080 | 0,049 | 0,157 | 0,141 | ,176^*^ | 0,095 | ,173^*^ | ,199^*^ | ,190^*^ |
| Superiorparietal | 0,029 | 0,057 | 0,038 | 0,106 | -0,032 | 0,120 | 0,125 | ,249^**^ | 0,064 | 0,132 | 0,116 | 0,133 |
| Superiortemporal | ,233^**^ | 0,077 | ,187^*^ | 0,112 | ,219^**^ | ,266^**^ | ,250^**^ | ,227^**^ | 0,135 | ,296^**^ | ,222^**^ | ,301^**^ |
| Supramarginal | ,170^*^ | 0,100 | ,173^*^ | ,220^**^ | ,173^*^ | ,227^**^ | 0,143 | ,189^*^ | 0,114 | ,263^**^ | ,209^**^ | ,268^**^ |
| Frontalpole | -,172^*^ | -0,056 | -0,121 | -0,075 | -0,135 | -0,122 | 0,003 | 0,000 | 0,023 | -0,099 | -0,101 | -0,109 |
| Temporalpole | ,184^*^ | 0,128 | ,184^*^ | ,217^**^ | ,184^*^ | ,270^**^ | 0,137 | ,213^**^ | 0,135 | ,267^**^ | ,250^**^ | ,283^**^ |
| Transversetemporal | ,210^**^ | 0,119 | ,196^*^ | 0,129 | ,161^*^ | ,169^*^ | ,195^*^ | ,208^**^ | 0,050 | ,231^**^ | ,192^*^ | ,239^**^ |
| Insula | ,229^**^ | 0,108 | ,241^**^ | 0,150 | ,246^**^ | ,233^**^ | ,207^*^ | ,195^*^ | ,169^*^ | ,303^**^ | ,227^**^ | ,308^**^ |
| Left Hemisfere Areas |  |  |  |  |  |  |  |  |  |  |  |  |
| Hippocampus | ,304^**^ | 0,147 | ,372^**^ | ,277^**^ | ,219^**^ | ,383^**^ | ,277^**^ | ,215^**^ | ,248^**^ | ,420^**^ | ,332^**^ | ,427^**^ |
| Caudalanteriorcingulate | 0,047 | -0,045 | -0,052 | -0,081 | -0,039 | -0,095 | 0,007 | 0,040 | 0,026 | 0,000 | -0,083 | -0,016 |
| Caudalmiddlefrontal | 0,032 | 0,027 | 0,079 | 0,097 | 0,012 | 0,049 | 0,074 | ,167^*^ | 0,131 | 0,138 | 0,058 | 0,129 |
| Cuneus | ,164^*^ | 0,106 | 0,154 | ,176^*^ | ,179^*^ | ,209^**^ | 0,155 | ,223^**^ | 0,095 | ,230^**^ | ,205^*^ | ,236^**^ |
| Entorhinal | ,237^**^ | 0,109 | ,303^**^ | 0,146 | 0,147 | ,283^**^ | ,187^*^ | ,158^*^ | ,172^*^ | ,290^**^ | ,238^**^ | ,299^**^ |
| Fusiform | ,212^**^ | ,175^*^ | ,263^**^ | ,255^**^ | ,216^**^ | ,327^**^ | ,222^**^ | ,329^**^ | ,279^**^ | ,389^**^ | ,310^**^ | ,395^**^ |
| Inferiorparietal | 0,055 | -0,061 | 0,095 | 0,120 | 0,094 | 0,155 | ,167^*^ | ,235^**^ | ,158^*^ | ,206^**^ | 0,071 | ,191^*^ |
| Inferiortemporal | 0,120 | 0,132 | 0,111 | ,253^**^ | 0,130 | ,224^**^ | ,194^*^ | ,282^**^ | ,172^*^ | ,286^**^ | ,231^**^ | ,294^**^ |
| Isthmuscingulate | ,230^**^ | 0,014 | ,233^**^ | 0,152 | 0,083 | 0,143 | ,186^*^ | 0,080 | ,181^*^ | ,246^**^ | 0,100 | ,226^**^ |
| Lateraloccipital | 0,126 | 0,030 | 0,108 | 0,080 | 0,038 | ,204^*^ | 0,057 | ,201^*^ | 0,121 | ,180^*^ | 0,150 | ,188^*^ |
| Lateralorbitofrontal | 0,039 | 0,034 | 0,155 | ,266^**^ | ,202^*^ | ,182^*^ | ,172^*^ | ,177^*^ | ,195^*^ | ,264^**^ | ,172^*^ | ,262^**^ |
| Lingual | 0,063 | 0,039 | 0,140 | ,191^*^ | 0,100 | ,170^*^ | 0,054 | ,261^**^ | 0,148 | ,231^**^ | 0,134 | ,220^**^ |
| Medialorbitofrontal | 0,058 | 0,083 | 0,113 | ,252^**^ | ,212^**^ | ,189^*^ | -0,004 | 0,126 | ,193^*^ | ,214^**^ | ,183^*^ | ,219^**^ |
| Middletemporal | 0,135 | ,158^*^ | 0,132 | ,238^**^ | 0,102 | ,211^**^ | ,201^*^ | ,332^**^ | ,179^*^ | ,285^**^ | ,235^**^ | ,294^**^ |
| Parahippocampal | ,228^**^ | 0,025 | ,231^**^ | ,160^*^ | 0,123 | ,279^**^ | ,256^**^ | ,216^**^ | 0,114 | ,285^**^ | ,196^*^ | ,284^**^ |
| Paracentral | 0,000 | 0,084 | 0,057 | 0,141 | 0,039 | ,168^*^ | 0,060 | ,170^*^ | 0,123 | 0,154 | ,163^*^ | ,165^*^ |
| Parsopercularis | 0,074 | -0,039 | 0,081 | 0,067 | 0,065 | 0,097 | ,171^*^ | 0,087 | 0,048 | 0,137 | 0,058 | 0,130 |
| Parsorbitalis | 0,046 | 0,024 | 0,064 | ,194^*^ | 0,063 | 0,086 | ,205^*^ | ,226^**^ | 0,100 | ,179^*^ | 0,084 | ,167^*^ |
| Parstriangularis | 0,132 | 0,099 | 0,067 | ,177^*^ | 0,091 | 0,106 | ,177^*^ | ,202^*^ | ,177^*^ | ,210^**^ | 0,137 | ,207^**^ |
| Pericalcarine | 0,109 | 0,093 | 0,033 | 0,132 | 0,060 | 0,095 | 0,055 | ,234^**^ | 0,091 | ,167^*^ | 0,124 | ,169^*^ |
| Postcentral | 0,107 | 0,086 | 0,086 | 0,125 | 0,063 | ,172^*^ | 0,113 | ,228^**^ | 0,129 | ,198^*^ | ,173^*^ | ,205^**^ |
| Posteriorcingulate | 0,145 | -0,065 | ,180^*^ | 0,103 | 0,066 | 0,086 | ,174^*^ | ,220^**^ | 0,085 | ,210^**^ | 0,017 | ,180^*^ |
| Precentral | 0,114 | -0,027 | 0,134 | 0,103 | -0,006 | 0,134 | ,216^**^ | ,263^**^ | 0,137 | ,211^**^ | 0,070 | ,190^*^ |
| Precuneus | ,233^**^ | 0,142 | ,278^**^ | ,202^*^ | ,162^*^ | ,309^**^ | ,234^**^ | ,248^**^ | ,174^*^ | ,323^**^ | ,281^**^ | ,332^**^ |
| Rostralanteriorcingulate | ,174^*^ | 0,060 | 0,117 | ,239^**^ | ,165^*^ | 0,102 | 0,048 | 0,154 | ,166^*^ | ,249^**^ | 0,118 | ,240^**^ |
| Rostralmiddlefrontal | 0,044 | 0,023 | 0,108 | 0,090 | 0,069 | 0,121 | 0,072 | 0,102 | ,194^*^ | ,168^*^ | 0,099 | ,166^*^ |
| Superiorfrontal | ,161^*^ | ,191^*^ | ,183^*^ | ,162^*^ | 0,134 | ,233^**^ | ,235^**^ | ,260^**^ | 0,130 | ,270^**^ | ,273^**^ | ,287^**^ |
| Superiorparietal | ,164^*^ | 0,065 | 0,149 | 0,146 | 0,075 | ,241^**^ | 0,130 | ,236^**^ | 0,132 | ,243^**^ | ,190^*^ | ,247^**^ |
| Superiortemporal | ,222^**^ | 0,064 | ,193^*^ | ,234^**^ | 0,129 | ,172^*^ | ,271^**^ | ,280^**^ | ,245^**^ | ,332^**^ | ,168^*^ | ,317^**^ |
| Supramarginal | 0,145 | 0,105 | ,235^**^ | ,193^*^ | 0,124 | ,194^*^ | ,260^**^ | ,269^**^ | ,191^*^ | ,308^**^ | ,199^*^ | ,303^**^ |
| Frontalpole | -0,099 | 0,056 | -0,013 | -0,107 | 0,015 | 0,114 | 0,057 | 0,003 | 0,014 | -0,016 | 0,097 | 0,014 |
| Temporalpole | 0,152 | 0,020 | ,196^*^ | ,236^**^ | 0,086 | ,185^*^ | ,161^*^ | ,189^*^ | 0,132 | ,242^**^ | 0,135 | ,235^**^ |
| Transversetemporal | ,264^**^ | 0,082 | ,213^**^ | 0,154 | 0,118 | ,188^*^ | ,277^**^ | 0,106 | ,175^*^ | ,259^**^ | ,188^*^ | ,259^**^ |
| Insula | ,163^*^ | 0,115 | ,174^*^ | ,164^*^ | ,175^*^ | ,245^**^ | ,218^**^ | ,204^*^ | 0,118 | ,269^**^ | ,241^**^ | ,283^**^ |

Abbrev. MAA = Middle-aged adult; OA = Older adult; Sec. Edu.: Secondary education; Univ. Edu.: University education. * p<0.05, ** p<0.001.

Supplemental table 2. Summary of the multiple linear regression results examining the influence of left hemisphere cortical volumes on various PD-CRS variables.

|  |  | Model 1 | | | Model 2 | | |
| --- | --- | --- | --- | --- | --- | --- | --- |
|  |  | B | 95% CI | β | B | 95% CI | β |
| \| Immediate_verbal_memory \| \| --- \| | Hippocampus |  |  |  | 0.014 | 0.002, 0,026 | 0.195** |
|  | Older Adults |  |  |  | -0.003 | -0.005, -0.001 | -0.257* |
|  | R-squared |  | 0.149** |  |  | 0.147* |  |
|  | Adjusted R-squared |  | 0.103 |  |  | 0.136 |  |
| Confrontation_naming | Fusiform | 0.005 | 0.001, 0.009 | 0.175* |  |  |  |
|  | R-squared |  | 0.030* |  |  | 0.093* |  |
|  | Adjusted R-squared |  | 0.024 |  |  | 0.057 |  |
| \| Sustained_attention \| \| --- \| | Hippocampus | 0.024 | 0.012, 0.036 | 0.375** | 0.020 | 0.008, 0.032 | 0.312* |
|  | R-squared |  | 0.202*** |  |  | 0.289*** |  |
|  | Adjusted R-squared |  | 0.158 |  |  | 0.228 |  |
| Working_memory | Medial Orbitofrontal |  |  |  | 0.021 | 0.007, 0.036 | 0.197* |
|  | Older Adults |  |  |  | -0.005 | -0.007, -0.004 | -0.418** |
|  | University Education |  |  |  | 0.002 | 0.000, 0.004 | 0.144* |
|  | R-squared |  | 0.151* |  |  | 0.281** |  |
|  | Adjusted R-squared |  | 0.105 |  |  | 0.266 |  |
| Spontaneous_clock_drawing | R-squared |  | 0.102* |  |  | 0.125* |  |
|  | Adjusted R-squared |  | 0.065 |  |  | 0.06 |  |
| Clock_copy | Hippocampus | 0.017 | 0.006, 0.029 | 0.285* | 0.017 | 0.007, 0.027 | 0.279** |
|  | Medial Orbitofrontal |  |  |  | 0.013 | 0.000, 0.026 | 0.143* |
|  | Older Adult |  |  |  | -0.002 | -0.004, -0.001 | -0.224* |
|  | R-squared |  | 0.226*** |  |  | 0.214*** |  |
|  | Adjusted R-squared |  | 0.178 |  |  | 0.199 |  |
| Delayed_verbal_memory | Hippocampus |  |  |  | 0.033 | 0.005,0.060 | 0.196* |
|  | Older Adult |  |  |  | -0.005 | -0.010, 0.00 | -0.173* |
|  | University Education |  |  |  | 0.007 | 0.001, 0.012 | 0.193* |
|  | R-squared |  | 0.142* |  |  | 0.150*** |  |
|  | Adjusted R-squared |  | 0.081 |  |  | 0.132 |  |
| Alternating_verbal_fluency | R-squared |  | 0.129* |  |  | 0.160* |  |
|  | Adjusted R-squared |  | 0.07 |  |  | 0.090 |  |
| Action_verbal_fluency | Medial Orbitofrontal |  |  |  | 0.018 | 0.002, 0.034 | 0.168* |
|  | Older Adults |  |  |  | -0.003 | -0.005, -0.001 | -0.266** |
|  | R-squared |  | 0.128* |  |  | 0.107*** |  |
|  | Adjusted R-squared |  | 0.061 |  |  | 0.096 |  |
| Frontosubcortical_score | Hippocampus | 0.022 | 0.014, 0.030 | 0.276** | 0.015 | 0.007, 0.022 | 0.271** |
|  | Medial Orbitofrontal | 0.015 | 0.004, 0.027 | 0.198* | 0.013 | 0.003, 0.024 | 0.169* |
|  | Older Adults |  |  |  | -0.003 | -0.005, -0.002 | -0.347** |
|  | R-squared |  | 0.217*** |  |  | 0.316*** |  |
|  | Adjusted R-squared |  | 0.207 |  |  | 0.302 |  |
| Posterior_cortical_score | Hippocampus |  |  |  | 0.007 | 0.002, 0.013 | 0.204** |
|  | Older Adults |  |  |  | -0.002 | -0.003, -0.001 | -0.295** |
|  | R-squared |  | 0.177* |  |  | 0.180*** |  |
|  | Adjusted R-squared |  | 0.126 |  |  | 0.169 |  |
| Total_pd_crs_score | Hippocampus | 0.017 | 0.010, 0,023 | 0.376** | 0.012 | 0.005, 0.018 | 0.270** |
|  | Medial Orbitofrontal | 0.012 | 0.002, 0,021 | 0.175* | 0.011 | 0.002, 0.020 | 0.165* |
|  | Transversetemporal | 0.024 | 0.000, 0,047 | 0.146* |  |  |  |
|  | Older Adults |  |  |  | -0.003 | -0.004, -0.001 | -0.342** |
|  | R-squared |  | 0.237*** |  |  | 0.313** |  |
|  | Adjusted R-squared |  | 0.222 |  |  | 0.299 |  |

The table only presents the coefficients and values for those independent variables and covariates that had a significant effect in the model. If no independent variable contributed significantly to the model but the model itself was significant, or if the model was not significant, only the R-squared and adjusted R-squared values are reported. Abbreviations: Model 1 refers to the multiple linear regression model in which only the cortical volume variables relevant to each analysis were included. Model 2 refers to the model adjusted for covariates, which included age, gender, educational level, and disease duration. Note: For Model 2 in the analysis of fronto-subcortical score, Model 2 in the analysis of posterior cortical score, Model 2 in the analysis of Immediate_verbal_memory, Model 2 in the analysis of delay verbal memory, Model 2 in the analysis of Spontaneous_clock_drawing, Model 2 in the analysis of action verbal fluency, and Models 1 and 2 n the analysis of Working Memory and total PD-CRS score, independent variables and covariates were entered into the model using the Stepwise method. P-values were adjusted for multiple comparisons using the Holm-Bonferroni correction. * p<0.05, ** p<0.01, *** p< 0.001.

*Supplemental table 3.* Summary of the multiple linear regression results examining the influence of right hemisphere cortical volumes on various PD-CRS variables.

|  |  | Model 1 | | | Model 2 | | |
| --- | --- | --- | --- | --- | --- | --- | --- |
|  |  | B | 95% CI | β | B | 95% CI | β |
| Immediate_verbal_memory | Isthmuscingulate | 0.041 | 0.012, 0.070 | 0.235* | 0.047 | 0.021, 0.073 | 0.269** |
|  | Hippocampus | 0.015 | 0.004, 0.026 | 0.225* |  |  |  |
|  | Older Adults |  |  |  | -0.03 | -0.005, -0.001 | -0.263** |
|  | R-squared |  | 0.157*** |  |  | 0.182** |  |
|  | Adjusted R-squared |  | 0.146 |  |  | 0.171 |  |
| Confrontation_naming | R-squared |  | 0.047* |  |  | 0.096** |  |
|  | Adjusted R-squared |  | 0.034 |  |  | 0.072 |  |
| Sustained_attention | Hippocampus | 0.022 | 0.001, 0.034 | 0.359* | 0.019 | 0.010, 0.029 | 0.315** |
|  | Older Adults |  |  |  | -0.002 | -0.004, 0.00 | -0.200* |
|  | Gender |  |  |  | -0.002 | -0.004, 0.00 | -0.172* |
|  | R-squared |  | 0.242*** |  |  | 0.251*** |  |
|  | Adjusted R-squared |  | 0.178 |  |  | 0.236 |  |
| Working_memory | Hippocampus | 0.020 | 0.010, 0.030 | 0.300** |  |  |  |
|  | Medial Orbitofrontal | 0.018 | 0.001, 0.034 | 0.160* |  |  |  |
|  | University Education |  |  |  | 0.002 | 0.000, 0.005 | 0.161* |
|  | Older Adults |  |  |  | -0.005 | -0.007,-0.004 | -0.433* |
|  | R-squared |  | 0.137*** |  |  | 0.243*** |  |
|  | Adjusted R-squared |  | 0.126 |  |  | 0.233 |  |
| Spontaneous_clock_drawing | R-squared |  |  |  |  |  |  |
|  | Adjusted R-squared |  |  |  |  |  |  |
| Clock_copy | Hippocampus | 0.020 | 0.009, 0.032 | 0.365* | 0.020 | 0.011, 0.029 | 0.364** |
|  | Older Adults |  |  |  | -0.002 | -0.004, 0.00 | -0.185* |
|  | R-squared |  | 0.235*** |  |  | 0.230*** |  |
|  | Adjusted R-squared |  | 0.171 |  |  | 0.220 |  |
| Delayed_verbal_memory | Isthmuscingulate |  |  |  | 0.095 | 0.033, 0.156 | 0.238** |
|  | Older Adults |  |  |  | -0.005 | -0.010, -0.001 | -0.189* |
|  | University Education |  |  |  | 0.006 | 0.001, 0.011 | 0.173* |
|  | R-squared |  | 0.144* |  |  | 0.170*** |  |
|  | Adjusted R-squared |  | 0.083 |  |  | 0.153 |  |
| Alternating_verbal_fluency | Precuneus | 0.021 | 0.010, 0.031 | 0.299** | 0.016 | 0.004, 0.027 | 0.223** |
|  | Older Adults |  |  |  | -0.003 | -0.006, -0.001 | -0.207* |
|  | R-squared |  | 0.089*** |  |  | 0.127*** |  |
|  | Adjusted R-squared |  | 0.084 |  |  | 0.115 |  |
| Action_verbal_fluency | Hippocampus | 0.013 | 0.002, 0.024 | 0.209* |  |  |  |
|  | Isthmuscingulate | 0.037 | 0.007, 0.067 | 0.195* | 0.041 | 0.014, 0.068 | 0.233** |
|  | University Education |  |  |  | 0.002 | 0.000, 0.005 | 0.160* |
|  | Older Adults |  |  |  | -0.002 | -0.004, 0.000 | -0.192* |
|  | R-squared |  | 0.186** |  |  | 0.163** |  |
|  | Adjusted R-squared |  | 0.175 |  |  | 0.146 |  |
| Frontosubcortical_score | Hippocampus | 0.018 | 0.010, 0.026 | 0.370** | 0.013 | 0.005, 0.021 | 0.262** |
|  | Isthmuscingulate | 0.030 | 0.010, 0.051 | 0.236** | 0.025 | 0.006, 0.045 | 0.196* |
|  | Older Adults |  |  |  | -0.002 | -0.004, -0.001 | -0.263** |
|  | University Education |  |  |  | 0.002 | 0.000, 0.003 | 0.155* |
|  | R-squared |  | 0.279*** |  |  | 0.372*** |  |
|  | Adjusted R-squared |  | 0.270 |  |  | 0.355 |  |
| Posterior_cortical_score | Hippocampus | 0.011 | 0.005, 0.018 | 0.359* | 0.010 | 0.004, 0.015 | 0.298** |
|  | Older Adults |  |  |  | -0.001 | -0.002, 0.000 | -0.240* |
|  | R-squared |  | 0.207** |  |  | 0.214*** |  |
|  | Adjusted R-squared |  | 0.141 |  |  | 0.204 |  |
| Total_pd_crs_score | Hippocampus | 0.016 | 0.009, 0,022 | 0.389** | 0.011 | 0.005, 0.018 | 0.280** |
|  | Isthmuscingulate | 0.024 | 0.006, 0,041 | 0.220* | 0.020 | 0.003, 0.036 | 0.184* |
|  | Older Adults |  |  |  | -0.002 | -0.003, -0.001 | -0.257** |
|  | University Education |  |  |  | 0.001 | 0.000, 0,003 | 0.142* |
|  | R-squared |  | 0.285*** |  |  | 0.369*** |  |
|  | Adjusted R-squared |  | 0.275 |  |  | 0.352 |  |

The table includes coefficients and values only for independent variables and covariates with a significant effect on the model. When no individual variable contributed significantly but the overall model was significant, or when the model was not significant, only the R-squared and adjusted R-squared values are shown. Model 1 refers to the multiple linear regression analysis including only cortical volume variables relevant to each specific analysis. Model 2 adjusts for covariates such as age, gender, educational level, and disease duration. For Model 2 in the analyses of sustained attention, clock copy, delay verbal memory, alternating verbal fluency, and posterior-cortical scores, as well as for Models 1 and 2 in working memory, alternating verbal fluency, Action_verbal_fluency, fronto-subcortical and total PD-CRS scores, variables were entered using the Stepwise method. P-values were adjusted for multiple comparisons using the Holm-Bonferroni correction. * p<0.05, ** p<0.01, *** p< 0.001.

Supplemental table 4. Descriptive table of controls. Values in parentheses represent the standard deviations of the means.

|  | CONTROLS | | | |
| --- | --- | --- | --- | --- |
| Age group | Young adults (30-55 years) | Middle-age adults (56-65 years) | Older adults (66-75 years) | Total |
| N | 12 | 26 | 7 | 45 |
| Male | 8 | 17 | 5 | 30 |
| Female | 4 | 9 | 2 | 15 |
| Age (mean) | 51,67 (1,96) | 60,88 (2,9) | 66,86 (1,06) | 59,36 (5,7) |
| Males (mean age) | 51,5 (1,6) | 60,29 (3,1) | 67 (1.22) | 59,07 (5,8) |
| Females (mean age) | 52 (2,82) | 62 (2,34) | 66,5 (0,7) | 59,93 (5,63) |
| Primary education | 4 | 6 | 4 | 14 |
| Secondary education | 6 | 11 | 2 | 19 |
| University education | 2 | 9 | 1 | 12 |
| Mean Intracraneal Volume | 1458,88 (177,94) | 1443,72 (154,75) | 1456,5 (86,99) | 1449,75 (150,35) |
| PDCRS total score Basal Visit (mean) | 102.66 (6.7) | 100.26 (7.87) | 94.85 (10.63) | 100.06 (8.26) |
| PDCRS total score 48M (mean) | 106.5 (10.73) | 102.52 (12.71) | 96.8 (8.22) | 102.61 (11.81) |
| PDCRS total score 60M (mean) | 108.5 (11.57) | 99.61 (12.73) | 100 (15.19) | 102.13 (13.21) |
| Subjects with basal a longitudinal data | 10 | 24 | 5 | 39 |

Supplemental table 5. Sensitivity Analyses of the multiple linear regression results examining the influence of **left hemisphere cortical volumes** on various PD-CRS variables.

|  |  | Model 1 | | | Model 2 | | |
| --- | --- | --- | --- | --- | --- | --- | --- |
|  |  | B | 95% CI | β | B | 95% CI | β |
| \| Immediate_verbal_memory \| \| --- \| | Hippocampus |  |  |  | 0.014 | 0.002, 0,026 | 0.195** |
|  | Older Adults |  |  |  | -0.003 | -0.005, -0.001 | -0.257* |
|  | R-squared |  | 0.149** |  |  | 0.147* |  |
|  | Adjusted R-squared |  | 0.103 |  |  | 0.136 |  |
| Confrontation_naming | Fusiform | 0,004 | 0.001, 0.006 | 0,253* | 0.002 | -0.003, 0.007 | 0.062 |
|  | R-squared |  | 0,064* |  |  | 0.093* |  |
|  | Adjusted R-squared |  | 0,058 |  |  | 0.057 |  |
| \| Sustained_attention \| \| --- \| | Hippocampus | 0,022 | 0.011, 0.033 | 0,386** | 0.016 | 0.005, 0.027 | 0.275 |
|  | R-squared |  | 0,234** |  |  | 0.331** |  |
|  | Adjusted R-squared |  | 0,191 |  |  | 0.273 |  |
| Working_memory | Medial Orbitofrontal |  |  |  | 0.021 | 0.007, 0.036 | 0.197* |
|  | Older Adults |  |  |  | -0.005 | -0.007, -0.004 | -0.418** |
|  | University Education |  |  |  | 0.002 | 0.000, 0.004 | 0.144* |
|  | R-squared |  | 0,151* |  |  | 0.281** |  |
|  | Adjusted R-squared |  | 0,105 |  |  | 0.266 |  |
| Spontaneous_clock_drawing | Hippocampus | 0.019 | 0.007, 0.031 | 0,322* |  |  |  |
|  | R-squared |  | 0,173** |  |  | 0,243** |  |
|  | Adjusted R-squared |  | 0,139 |  |  | 0,189 |  |
| Clock_copy | Hippocampus | 0.015 | 0.005, 0.0024 | 0,292* | 0.014 | 0.005, 0.024 | 0.272** |
|  | Fusiform |  |  |  | 0.009 | 0.002, 0.016 | 0.230* |
|  | R-squared |  | 0.289** |  |  | 0,201** |  |
|  | Adjusted R-squared |  | 0.244 |  |  | 0,190 |  |
| Delayed_verbal_memory | Hippocampus |  |  |  | 0.042 | 0.011 | 0.292** |
|  | Transversetemporal |  |  |  | 0.117 | 0.040 | 0.227* |
|  | R-squared |  | 0.192** |  |  | 0.173** |  |
|  | Adjusted R-squared |  | 0.134 |  |  | 0.162 |  |
| Alternating_verbal_fluency | R-squared |  | 0.161* |  |  | 0,181* |  |
|  | Adjusted R-squared |  | 0.102 |  |  | 0,112 |  |
| Action_verbal_fluency | Hippocampus |  |  |  | 0.013 | 0.002, 0.024 | 0.192* |
|  | Medial Orbitofrontal |  |  |  | 0.016 | 0.001, 0.31 | 0.156* |
|  | Older Adults |  |  |  | -0.002 | -0.004, 0.00 | -0.203* |
|  | University education |  |  |  | 0.003 | 0.00, 0.005 | 0.177* |
|  | R-squared |  | 0.160* |  |  | 0.200** |  |
|  | Adjusted R-squared |  | 0.095 |  |  | 0.179 |  |
| Frontosubcortical_score | Hippocampus | 0.022 | 0.014, 0.030 | 0.276** | 00015 | 0.007,0.022 | 0.278** |
|  | Medial Orbitofrontal | 0.015 | 0.004, 0.027 | 0.198* |  |  |  |
|  | Older Adults |  |  |  | -0.003 | -0.005, -0.002 | -0.316** |
|  | University Education |  |  |  | 0.002 | 0.00, 0.003 | 0.165* |
|  | R-squared |  | 0.217*** |  |  | 0.348** |  |
|  | Adjusted R-squared |  | 0.207 |  |  | 0.334 |  |
| Posterior_cortical_score | Hippocampus | 0.007 | 0.003, 0.013 | 0,318* | 0.007 | 0.003, 0.011 | -0.294** |
|  | Older Adults |  |  |  | -0.001 | -0.002, 0.00 | -0.271** |
|  | R-squared |  | 0.246** |  |  | 0.226*** |  |
|  | Adjusted R-squared |  | 0.198 |  |  | 0.216 |  |
| Total_pd_crs_score | Hippocampus | 0.016 | 0.010, 0.022 | 0.379* | 0.012 | 0.006, 0.018 | 0.289** |
|  | Medial Orbitofrontal | 0.011 | 0.002, 0.020 | 0.183** | 0.010 | 0.001, 0.018 | 0.158* |
|  | Transversetemporal | 0.027 | 0.005, 0.050 | 0.178* |  |  |  |
|  | University Education |  |  |  |  |  |  |
|  | Older Adults |  |  |  | -0.003 | -0.004, -0.001 | -0355** |
|  | R-squared |  | 0.260** |  |  | 0.337** |  |
|  | Adjusted R-squared |  | 0.245 |  |  | 0.324 |  |
|  |  |  |  |  |  |  |  |

*Supplemental table 6.* Sensitivity Analyses of the multiple linear regression results examining the influence of **right hemisphere cortical volumes** on various PD-CRS variables.

|  |  | Model 1 | | | Model 2 | | |
| --- | --- | --- | --- | --- | --- | --- | --- |
|  |  | B | 95% CI | Β | B | 95% CI | β |
| Immediate_verbal_memory | Isthmuscingulate | 0.043 | 0.015, 0.071 | 0.251* | 0.047 | 0.021, 0.073 | 0.269** |
|  | Hippocampus | 0.016 | 0.005, 0.026 | 0.251* |  |  |  |
|  | R-squared |  | 0,182** |  | -0.03 | -0.005, -0.001 | -0.263** |
|  | Adjusted R-squared |  | 0,171 |  |  | 0.182** |  |
| Confrontation_naming | Hippocampus | 0.005 | 0.002, 0.009 | 0,273* |  |  |  |
|  | Older Adults |  |  |  | -0.001 | -0.001, 0.00 | -0.246* |
|  | R-squared |  | 0.099** |  |  | 0.152** |  |
|  | Adjusted R-squared |  | 0.087 |  |  | 0.129 |  |
| Sustained_attention | Hippocampus | 0.023 | 0.012, 0.033 | 0.433* | 0.019 | 0.011, 0.027 | 0.356** |
|  | Older Adults |  |  |  | -0.002 | -0.004, -0.001 | -0.245** |
|  | Gender |  |  |  | -0.002 | -0.003,-0.001 | -0.192** |
|  | R-squared |  | 0.284** |  |  | 0.338** |  |
|  | Adjusted R-squared |  | 0.222 |  |  | 0.325 |  |
| Working_memory | Hippocampus | 0.020 | 0.010, 0.30 | 0.300** |  |  |  |
|  | Medial Orbitofrontal | 0.018 | 0.001, 0.034 | 0.160* |  |  |  |
|  | University Education |  |  |  | 0.002 | 0.000, 0.005 | 0.161* |
|  | Older Adults |  |  |  | -0.005 | -0.007,-0.004 | -0.433* |
|  | R-squared |  | 0.137** |  |  | 0.243*** |  |
|  | Adjusted R-squared |  | 0.126 |  |  | 0.233 |  |
| Spontaneous_clock_drawing | Hippocampus | 0.018 | 0.006, 0.030 | 0.317* |  |  |  |
|  | R-squared |  | 0,188** |  |  | 0.231* |  |
|  | Adjusted R-squared |  | 0,118 |  |  | 0.159 |  |
| Clock_copy | Hippocampus | 0.020 | 0.011, 0.030 | 0.435* | 0.020 | 0.012, 0.027 | 0.435** |
|  | Precuneus |  |  |  | 0.006 | 0.00, 0.013 | 0,159* |
|  | R-squared |  | 0.300** |  |  | 0,269** |  |
|  | Adjusted R-squared |  | 0.240 |  |  | 0,260 |  |
| Delayed_verbal_memory | Hippocampus | 0.034 | 0.006, 0.063 | 0.263* | 0.035 | 0.013, 0.057 | 0.268* |
|  | Isthmuscingulate |  |  |  | 0.070 | 0.012, 0.128 | 0.205* |
|  | R-squared |  | 0.197** |  |  | 0.168** |  |
|  | Adjusted R-squared |  | 0.139 |  |  | 0.156 |  |
| Alternating_verbal_fluency | Inferior Parietal | 0.017 | 0.008, 0.025 | 0.306* | 0.013 | 0.005, 0.022 | 0.244** |
|  | Older Adults |  |  |  | -0.003 | -0.005,-0.001 | -0.205* |
|  | R-squared |  | 0.093** |  |  | 0.132** |  |
|  | Adjusted R-squared |  | 0.087 |  |  | 0.120 |  |
| Action_verbal_fluency | Hippocampus | 0.014 | 0.003, 0.024 | 0.221* |  |  |  |
|  | Isthmuscingulate | 0.046 | 0.019, 0.074 | 0.279* | 0.052 | 0.027, 0.076 | 0.310** |
|  | Older Adults |  |  |  | -0.003 | -0.005, -0.002 | -0.277** |
|  | R-squared |  | 0.186*** |  |  | 0.219** |  |
|  | Adjusted R-squared |  | 0.175 |  |  | 0.209 |  |
| Frontosubcortical_score | Hippocampus | 0.018 | 0.011, 0.025 | 0.393** | 0.013 | 0.005, 0.020 | 0.273** |
|  | Isthmuscingulate | 0.027 | 0.007,0.046 | 0.218* | 0.021 | 0.003, 0.039 | 0.173* |
|  | Older Adults |  |  |  | -0.003 | -0.004, -0.001 | -0.293** |
|  | University Education |  |  |  | 0.002 | 0.00, 0.003 | 0.167* |
|  | R-squared |  | 0.288** |  |  | 0.399** |  |
|  | Adjusted R-squared |  | 0.270 |  |  | 0.382 |  |
| Posterior_cortical_score | Hippocampus | 0.009 | 0.004, 0.015 | 0,385** | 0.008 | 0.004, 0.012 | 0.308** |
|  | Older Adults |  |  |  | -0.001 | -0.002, 0.00 | -0.265* |
|  | R-squared |  | 0.246** |  |  | 0.240** |  |
|  | Adjusted R-squared |  | 0.181 |  |  | 0.230 |  |
| Total_pd_crs_score | Hippocampus | 0.016 | 0.010, 0.022 | 0.413** | 0.011 | 0.005, 0.017 | 0.295** |
|  | Isthmuscingulate | 0.020 | 0.004, 0.037 | 0.203* | 0.016 | 0.001, 0.032 | 0.163* |
|  | Older Adults |  |  |  | -0.002 | -0.003, -0.001 | -0.279** |
|  | University Education |  |  |  | 0.001 | 0.00, 0.002 | 0.151* |
|  | R-squared |  | 0.296*** |  |  | 0.393*** |  |
|  | Adjusted R-squared |  | 0.286 |  |  | 0.376 |  |

Supplemental table 7. Sensitivity analyses of the multiple linear regression results examining the influence of left hemisphere cortical volumes on various PD-CRS variables considering age as a continuous variable.

|  |  |  | Model 2 | | |
| --- | --- | --- | --- | --- | --- |
|  |  |  | B | 95% CI | β |
| \| Immediate_verbal_memory \| \| --- \| |  |  |  |  |  |
|  |  | Age | -0.0002 | -0.0003, -0.0001 | -0.375** |
|  |  | R-squared |  | 0.141* |  |
|  |  | Adjusted R-squared |  | 0.135 |  |
| Confrontation_naming |  | Age | -0.00007 | -0.001, 0 | -0.218* |
|  |  | Time of disease evolution | -0.00001 | -0.00001, 0 | -0.169 |
|  |  | R-squared |  | 0.097* |  |
|  |  | Adjusted R-squared |  | 0.067 |  |
| \| Sustained_attention \| \| --- \| |  | Hippocampus | 0.017 | 0.007, 0.028 | 0.261** |
|  |  | Gender | -0.002 | -0.004, -0.001 | -0.196* |
|  |  | Age | -0.0001 | -0.00022, -0.00002 | -0.211 |
|  |  | R-squared |  | 0.223 |  |
|  |  | Adjusted R-squared |  | 0.207 |  |
| Working_memory |  | Medial Orbitofrontal | 0.025 | 0.009, 0.040 | 0.277* |
|  |  | Age | -0.0002 | -0.0003, -0.0001 | -0.323** |
|  |  | University Education | 0.002 | 0.00007, 0.004 | 0.144* |
|  |  | R-squared |  | 0.213** |  |
|  |  | Adjusted R-squared |  | 0.198 |  |
| Spontaneous_clock_drawing |  | R-squared |  | 0.108* |  |
|  |  | Adjusted R-squared |  | 0.05 |  |
| Clock_copy |  | Hippocampus | 0.023 | 0.014, 0.033 | 0.279** |
|  |  | Medial Orbitofrontal | 0.015 | 0.001, 0.023 | 0.161* |
|  |  | R-squared |  | 0.172*** |  |
|  |  | Adjusted R-squared |  | 0.162 |  |
| Delayed_verbal_memory |  | Hippocampus | 0.037 | 0.011,0.062 | 0.220* |
|  |  | University Education | 0.006 | 0.001, 0.012 | 0.188* |
|  |  | R-squared |  | 0.154*** |  |
|  |  | Adjusted R-squared |  | 0.137 |  |
| Alternating_verbal_fluency |  | R-squared |  | 0.149* |  |
|  |  | Adjusted R-squared |  | 0.078 |  |
| Action_verbal_fluency |  | Fusiform | 0.015 | 0.006, 0.023 | 0.267* |
|  |  | University Education | 0.003 | 0.001, 0.005 | 0.192** |
|  |  | R-squared |  | 0.115*** |  |
|  |  | Adjusted R-squared |  | 0.103 |  |
| Fronto-subcortical score ^&^ |  | Hippocampus | 0.013 | 0.005, 0.021 | 0.250** |
|  |  | Medial Orbitofrontal | 0.015 | 0.004, 0.025 | 0.185** |
|  |  | Age | -0.0001 | (-0.0002, -0.00008) | -0.325** |
|  |  | R-squared |  | 0.291*** |  |
|  |  | Adjusted R-squared |  | 0.277 |  |
| Posterior-cortical score ^&^ |  | Hippocampus | 0.007 | 0.001, 0.013 | 0.206* |
|  |  | Age | -0,00007 | (-0.0001, -0.00002 | -0.236* |
|  |  | Time of disease evolution | -0,00007 |  | -0.158* |
|  |  | R-squared |  | 0.185*** |  |
|  |  | Adjusted R-squared |  | 0.169 |  |
| Total PD-CRS score ^&^ |  | Hippocampus | 0.011 | 0.005, 0.018 | 0.257** |
|  |  | Medial Orbitofrontal | 0.012 | 0.004, 0.021 | 0.190* |
|  |  |  |  |  |  |
|  |  | Age | -0.0001 | -0.0002, -0.00006 | -0.322** |
|  |  | R-squared |  | 0.297** |  |

The table only presents the coefficients and values for those independent variables and covariates that had a significant effect in the model. If no independent variable contributed significantly to the model but the model itself was significant, or if the model was not significant, only the R-squared and adjusted R-squared values are reported. To maintain precision and readability, coefficients and CI are reported up to five decimal places when their absolute value is less than 0.01 Model 1 refers to the multiple linear regression model in which only the cortical volume variables relevant to each analysis were included. Model 2 refers to the model adjusted for covariates, which included age, gender, educational level, and disease duration. Note: For Model 2 in the analysis of fronto-subcortical score, Model 2 in the analysis of posterior cortical score, Model 2 in the analysis of Immediate_verbal_memory, Model 2 in the analysis of delay verbal memory, Model 2 in the analysis of Spontaneous_clock_drawing, Model 2 in the analysis of action verbal fluency, and Models 1 and 2 n the analysis of Working Memory and total PD-CRS score, independent variables and covariates were entered into the model using the Stepwise method. P-values were adjusted for multiple comparisons using the Holm-Bonferroni correction. * p<0.05, ** p<0.01, *** p< 0.001.

Supplemental table 8. Sensitivity analyses of the multiple linear regression results examining the influence of right hemisphere cortical volumes on various PD-CRS variables, considering age as a continuous variable.

|  |  | Model 2 | | |
| --- | --- | --- | --- | --- |
|  |  | B | 95% CI | Β |
| Immediate_verbal_memory | Isthmuscingulate | 0.040 | 0.013, 0.067 | 0.230** |
|  | Age | -0.00018 | -0.00028, -0.00008 | -0.283** |
|  | R-squared |  | 0.185** |  |
|  | Adjusted R-squared |  | 0.175 |  |
| Confrontation_naming | R-squared |  | 0.100** |  |
|  | Adjusted R-squared |  | 0.076 |  |
| Sustained_attention | Hippocampus | 0.020 | 0.010, 0.030 | 0.329** |
|  | Precuneus | 0.008 | 0.0001, 0.016 | -0.162* |
|  | Gender | -0.003 | -0.004, -0.001 | -0.225* |
|  | R-squared |  | 0.242*** |  |
|  | Adjusted R-squared |  | 0.227 |  |
| Working_memory |  |  |  |  |
|  | Medial Orbitofrontal | 0.018 | 0.002,0.034 | 0.168* |
|  | University Education | 0.002 | 0.000, 0.005 | 0.163* |
|  | Age | -0.0002 | -0.0003,-0.0001 | -0.303* |
|  | R-squared |  | 0.190*** |  |
|  | Adjusted R-squared |  | 0.174 |  |
| Spontaneous_clock_drawing | R-squared |  |  |  |
|  | Adjusted R-squared |  |  |  |
| Clock_copy | Hippocampus | 0.026 | 0.018, 0.034 | 0.449** |
|  |  |  |  |  |
|  | R-squared |  | 0.2020*** |  |
|  | Adjusted R-squared |  | 0.196 |  |
| Delayed_verbal_memory | Isthmuscingulate | 0.079 | 0.011, 0.147 | 0.198* |
|  | Hippocampus | 0.029 | 0.003, 0.055 | 0.189* |
|  | University Education | 0.007 | 0.002, 0.012 | 0.208* |
|  | R-squared |  | 0.166*** |  |
|  | Adjusted R-squared |  | 0.149 |  |
| Alternating_verbal_fluency | Precuneus | 0.021 | 0.010, 0.032 | 0.302** |
|  | R-squared |  | 0.091*** |  |
|  | Adjusted R-squared |  | 0.085 |  |
| Action_verbal_fluency | Hippocampus | 0.013 | 0.002, 0.024 | 0.198* |
|  | Isthmuscingulate | 0.033 | 0.003, 0.062 | 0.186* |
|  | University Education | 0.003 | 0.001, 0.005 | 0.184* |
|  |  |  |  |  |
|  | R-squared |  | 0.153** |  |
|  | Adjusted R-squared |  | 0.136 |  |
| Fronto-subcortical score ^&^ | Hippocampus | 0.018 | 0.010, 0.025 | 0.365** |
|  |  |  |  |  |
|  | Age | -0.00008 | -0.00016, -0.00001 | -0.217* |
|  | University Education | 0.002 | 0.000, 0.003 | 0.174* |
|  | R-squared |  | 0.315*** |  |
|  | Adjusted R-squared |  | 0.301 |  |
| Posterior-cortical score ^&^ | Hippocampus | 0.010 | 0.005, 0.016 | 0.318** |
|  | Age | -0.00005 | -0.0001, 0.00000 | -0.183* |
|  | R-squared |  | 0.196*** |  |
|  | Adjusted R-squared |  | 0.186 |  |
| Total PD-CRS score ^&^ | Hippocampus | 0.015 | 0.009, 0.022 | 0.381** |
|  |  |  |  |  |
|  | Age | -0.00007 | -0.0001, -0.000 | -0.212* |
|  | University Education | 0.001 | 0.0002, 0,003 | 0.161* |
|  | R-squared |  | 0.320*** |  |
|  | Adjusted R-squared |  | 0.307 |  |

The table includes coefficients and values only for independent variables and covariates with a significant effect on the model. When no individual variable contributed significantly but the overall model was significant, or when the model was not significant, only the R-squared and adjusted R-squared values are shown. To maintain precision and readability, coefficients and CI are reported up to five decimal places when their absolute value is less than 0.01 Model 1 refers to the multiple linear regression analysis including only cortical volume variables relevant to each specific analysis. Model 2 adjusts for covariates such as age, gender, educational level, and disease duration. For Model 2 in the analyses of sustained attention, clock copy, delay verbal memory, alternating verbal fluency, and posterior-cortical scores, as well as for Models 1 and 2 in working memory, alternating verbal fluency, Action_verbal_fluency, fronto-subcortical and total PD-CRS scores, variables were entered using the Stepwise method. P-values were adjusted for multiple comparisons using the Holm-Bonferroni correction. * p<0.05, ** p<0.01, *** p< 0.001.
